# Metformin exhibits preventive and therapeutic efficacy against experimental cystic echinococcosis

**DOI:** 10.1371/journal.pntd.0005370

**Published:** 2017-02-09

**Authors:** Julia A. Loos, Valeria A. Dávila, Christian R. Rodrígues, Romina Petrigh, Jorge A. Zoppi, Fernando A. Crocenzi, Andrea C. Cumino

**Affiliations:** 1 Laboratorio de Zoonosis Parasitarias, Departamento de Biología, Facultad de Ciencias Exactas y Naturales, Universidad Nacional de Mar del Plata (UNMdP), Funes 3350, Nivel Cero, Mar del Plata, Argentina; 2 Consejo Nacional de Investigaciones Científicas y Técnicas (CONICET), Buenos Aires, Argentina; 3 Departamento de Química, Facultad de Ciencias Exactas y Naturales, Universidad Nacional de Mar del Plata (UNMdP), Funes 3350, Nivel 2, Mar del Plata, Argentina; 4 Servicio de Patología, Hospital Privado de Comunidad (HPC), Córdoba 4545, Nivel 3, Mar del Plata, Argentina; 5 Instituto de Fisiología Experimental (IFISE), Universidad Nacional de Rosario (UNR), Suipacha 570, Rosario, Argentina; Istituto Superiore di Sanità, ITALY

## Abstract

Metformin (Met) is an anti-hyperglycemic and potential anti-cancer agent which may exert its anti-proliferative effects via the induction of energetic stress. In this study we investigated the *in vitro* and *in vivo* efficacy of Met against the larval stage of *Echinococcus granulosus*. Metformin showed significant dose- and time-dependent killing effects on *in vitro* cultured protoscoleces and metacestodes. Notably, the combination of Met together with the minimum effective concentration of ABZSO had a synergistic effect after days 3 and 12 on metacestodes and protoscoleces, respectively. Oral administration of Met (50 mg/kg/day) in *E*. *granulosus*-infected mice was highly effective in reducing the weight and number of parasite cysts, yet its combination with the lowest recommended dose of ABZ (5 mg/kg/day) was even more effective. Coincidentally, intracystic Met accumulation was higher in animals treated with both drugs compared to those administered Met alone. Furthermore, the safe plant-derived drug Met exhibited remarkable chemopreventive properties against secondary hydatidosis in mice. In conclusion, based on our experimental data, Met emerges as a promising anti-echinococcal drug as it has proven to efficiently inhibit the development and growth of the *E*. *granulosus* larval stage and its combination with ABZ may improve the current anti-parasitic therapy.

## Author summary

Cystic echinococcosis is a worldwide zoonosis of public health concern and economic significance caused by infection with the larval stage of the cestode *Echinococcus granulosus*. Chemotherapy treatment for this disease has had limited effectiveness thus far, which is why it is a dire need to find new drugs for its treatment. In order to survive, *E*. *granulosus* must compete with the host for the same metabolic resources. However, the cell energy is a commodity that these organisms cannot directly obtain from the host. Therefore, our treatment strategy was to interfere with the energy-generating and cell proliferation mechanisms in the larval stage of this cestode. In this study we focus on metformin, an anti-hyperglycemic and anti-proliferative drug, which exhibits considerable *in vitro* and *in vivo* activity against *E*. *granulosus* metacestodes. In experimentally infected mice, metformin-chemopreventive effect was evidenced and the combination of this drug with low doses of albendazole improved the anti-parasitic therapy results in late stage of cyst development. Therefore, the effects of these two drugs against *E*. *granulosus* warrant further investigations, in comparison to the current monotherapy of choice in humans carried out with albendazole.

## Introduction

Cystic echinococcosis (CE), also called hydatid disease or hydatidosis, is a neglected zoonotic disease caused by the infection with the larval stage of the cestode *Echinococcus granulosus* [1]. The greatest prevalence of this disease is found in countries of the temperate zones, and it is an endemic disease in some parts of the world, such as South America, Asia, Australia and North Africa [2]. It has been estimated that human CE results in the loss of 1–3 million disability-adjusted life years (DALYs) and that up to $2 billion are annually lost in the livestock industry [3]. The mortality rate due to CE is about 2–4%, but it may increase considerably if medical treatment is not suitable [4].

The life cycle of *E*. *granulosus* is complex and involves two mammalian hosts. The adult cestode inhabits the small intestine of a carnivorous definitive host (usually dogs) and produces eggs, which are released into the environment and may then be ingested by an intermediate host (ungulates, or humans). Metacestodes or hydatid cysts proliferate asexually in the intermediate host and produce protoscoleces from the inner germinal layer [5]. The liver is the most commonly affected organ in patients infected with CE, followed by the lungs and, less frequently by organs such as spleen, kidneys, heart, bones, and central nervous system [2]. The growth of hydatid cysts is usually slow and asymptomatic, and clinical manifestations are related to compression of the involved organs. However, cyst rupture may lead to anaphylactic reactions as well as dissemination and/or recurrence of the infection [6].

The treatment options for CE are PAIR (Puncture, Aspiration, Injection, Reaspiration), surgery and chemotherapy, and the choice is often based on cyst characteristics as well as availability of medical experts and equipment. Surgery is accompanied by pre- and post-operative chemotherapy and, in inoperable cases, chemotherapy is the only alternative [4]. Despite the fact that the benzimidazoles (BMZs) albendazole (ABZ) and mebendazole (MBZ) are currently the only two drugs licensed for the treatment of CE, the cure rate of both drugs in treatment of CE was reported to be only about 30% [7]. The unsatisfactory results of the oral chemotherapy with BMZs are usually attributed to their poor absorption rate, resulting in low drug levels in the plasma and thus in the hydatid cysts [8]. For this reason, and the fact that the adverse effects of BMZ seem to be inevitable under therapeutic doses, it is necessary to find other alternatives for chemotherapy against CE, and to focus on new drugs with higher anthelminthic activity against *E*. *granulosus*.

Metformin (Met) is an anti-hyperglycemic agent widely used for the treatment of type II diabetes which shows good oral bioavailability (50–60%) and a favorable safety profile [9, 10]. Notably, this drug also has anti-proliferative properties on cancer cells, which it may exert both indirectly, through the systemic reduction of insulin levels in diabetic patients, and directly, via the induction of energetic stress, in non-diabetic and diabetic patients [11–13]. Direct cellular effects of Met involve inhibition of ATP production, activation of AMPK, and consequent inhibition of TORC1 (Target Of Rapamycin Complex 1), which couples protein synthesis to external growth factors and intracellular energy stores [12, 14]. Recently, we have reported that rapamycin, an inhibitor of TOR, is an effective *in vitro* anti-echinococcal agent and autophagy inducer, allowing the identification of TORC1-controlled events in this cestode [15]. In addition, we have demonstrated that, under *in vitro* conditions, both larval forms of *E*. *granulosus* are susceptible to Met and that this drug indirectly activates Eg-AMPK (AMP activated protein kinase), as a consequence of cellular energy charge depletion [16]. The Met pharmacokinetics is regulated by transporters of the major facilitator superfamily (MFS) through a balance between uptake and expulsion mechanisms. The first ones are dependent on expression of solute carrier family 22 members -SLC22- (OCT 1, 2, and 3 and OCTN1), while the second ones are dependent on expression of multidrug and toxin extrusion proteins (MATE1 and MATE 2) [12, 17].

Here we demonstrate that Met alone or in combination with low concentration of ABZ is effective against the larval stage of *E*. *granulosus* in the murine CE infection model, and we discuss the Met-intracyst accumulation.

## Methods

### Chemicals

Metformin (1,1-dimethylbiguanide hydrochloride) was obtained from Sigma-Aldrich (USA) and ABZ and ABZSO were kindly provided by C. Salomon (National University of Rosario, Argentina). For *in vitro* assays, Met and ABZSO were kept as a 100 mM and a 100 μM stock solution in water and in dimetyl sulfoxide (DMSO), respectively, and added to the medium either separately or in combination. For *in vivo* experiments, aqueous solution of Met and oil solution of ABZ (corn oil, Sigma-Aldrich) were prepared every 2 days from solid drug and maintained under refrigeration (3–5°C).

### Ethics statement

Animal procedures and management protocols were carried out in accordance with the National Health Service and Food Quality (SENASA) guidelines, Argentina, and with the 2011 revised form of The Guide for the Care and Use of Laboratory Animals published by the U.S. National Institutes of Health. Experimental protocols were evaluated and approved by the Animal Experimental Committee at the Faculty of Exact and Natural Sciences, Mar del Plata University (permit number: 2555-08-15).

### *In vitro* drug testing assays on larval stage of *E*. *granulosus*

Protoscoleces were removed aseptically from hydatid cysts of infected cattle slaughtered in the Liminal abattoir (official number: 3879) located in the Southeast of Buenos Aires, Argentina. Viable and morphologically intact protoscoleces (*n* = 3,000) were cultured using medium 199 (Gibco) supplemented with glucose (4 mg/ml) and antibiotics (penicillin, streptomycin and gentamicin 100 μg/ml) in 24-well culture plates under normal atmospheric conditions as we described in detail previously [15]. Moreover, from10 to 20 *E*. *granulosus* murine cysts per replica were incubated in Leighton tubes under the same culture conditions as described for protoscoleces [16]. *In vitro* protoscolex and metacestode treatments were performed with 1 and 5 mM Met, 2.5 μM ABZSO (equivalent to 0.84 μg/ml), and the combination of 1 and 5 mM Met plus 2.5 μM ABZSO for 7 (metacestodes) and 27 (protoscoleces) days [16]. Parasites incubated in culture medium containing DMSO were used as controls. *In vitro* protoscolex cultures were kept at 37°C with medium changes every 3 days. At those time points, the protoscolex viability was determined by the methylene blue exclusion test (at least 100 protoscoleces per replica were counted each time). The metacestode viability was assessed daily by trypan blue staining of detached germinal layers. Each experiment was performed in triplicates and repeated three times. All of the experiments were carried out until that the viability of the control was lower than 90% or all treated parasites were dead. For molecular assays, protoscoleces and metacestodes were cultured in presence or absence of 1 or 5 mM Met for 48 h and stored at −80°C until experimental use.

### Experimental animals and determination of efficacy of *in vivo* treatments

Healthy female CF-1 mice (30–35 g of 8 weeks old) supplied by the SENASA, Mar del Plata, were acclimatized for one week before initiation of the experiment. Mice were infected by intraperitoneal infection with 1,000 protoscoleces in 0.5 ml of medium 199 to produce experimental secondary hydatid disease [16]. The animals were maintained in standard polyethylene cages (five mice per cage), under controlled laboratory conditions (temperature 20±2°C, 12 hour light/12 hour dark with lights off at 8.00 p.m., 50±5% humidity). Food and water were provided *ad libitum*. Every 3 days, animals were placed into a clean cage with fresh sawdust. All the pharmacological treatments were performed by intragastric administration of a drug-aqueous suspension (0.3 ml/animal). At the end of experiments, mice were euthanized by cervical dislocation and previous anesthesia with ketamine–xylazine (50 mg/kg/mouse– 5 mg/kg/mouse). All efforts were made to minimize suffering. Minimum number of animals was used in each experiment. At necropsy, the peritoneal cavity was opened, the hydatid cysts were carefully recorded, and their weights were determined for each animal. The efficacy of treatments was calculated using the following formula: 100 x {(mean cyst weight of control group)–(mean cyst weight of treated group)}/ (mean cyst weight of control group). In addition, samples were processed for scanning electron microscopy (SEM) with a JEOL JSM-6460LV electron microscope and for transmission electron microscopy (TEM) with a JEM 1200 EX II (JEOL Ltd., Tokio, Japan) microscopy as previously described [15]. Further, histological examination and evaluation of hepatic fibrosis in mice were carried out. Liver samples were fixed in formalin, embedded in paraffin and 5 μm thick sections were stained with hematoxylin and eosin and Masson’s trichrome [18]. These specimens were photographed and the collagen depositions were quantified from 3–5 images of each sample using Image-J software.

### Therapeutic effectiveness of metformin and its combination with albendazole

At 4 months p.i., mice were randomly assigned into 4 groups of 10 animals each. Drugs were applied by per oral gavage daily for 60 days as follows: control group (receiving corn oil as a placebo), ABZ at 5 mg/kg/day, Met at 50 mg/kg/day, and a combination of ABZ (5 mg/kg/day) plus Met (50 mg/kg/day). At the end of treatment period, animals were euthanized, necropsy was carried out immediately thereafter, and Met content was determined from hydatid liquid as described below.

### Chemoprophylactic efficacy of metformin

At the time point of infection, 20 CF-1 mice were allocated into 2 experimental groups (10 animals/group) as untreated control group (corn oil) and Met-treated group (50 mg/kg/day). The treatment was performed daily by 60 days. Four months after infection, mice were euthanized and necropsied.

### Determination of metformin levels

Two spectrophotometric methods were used for the estimation of intracystic Met concentrations, based on the reaction in alkaline medium of the primary amino group of Met with ninhydrin or hydrogen peroxide, to form a violet (570 nm) or yellow (400 nm) chromogen, respectively [19, 20]. Standard curves were prepared using a double spectrophotometer (Shimatzu-UV-100) and different concentrations of pure Met solutions (10–100 μg of drug), which obeyed Beer´s law in the range of 5–20 μg/ml. Hydatidic cyst fluid was extracted immediately after necropsy from samples of untreated or treated mice, the precipitated protein (at 2000×g for 15 min) was removed and the supernatants were stored at -20°C until colorimetric analysis.

### Genomic identification of *Echinococcus* SLC-like transporters

Given that Met is positively charged at physiological pH and its cellular transport depends on cationic transporters, BLASTp search for solute carrier transporter family 22 (SLC22) homologs in the *E*. *granulosus* genome database (http://www.sanger.ac.uk/Projects/Echinococcus, [21]) was carried out using *Mus musculus* and *Homo sapiens* orthologs as queries. Orthologs were selected based on reciprocal best BLAST hits [25, 26] on an E-value cut-off of 10^−25^ and on the presence of the characteristic domains in each deduced amino acid sequence. Sequence alignments were generated with the CLUSTALX software program and modeling of secondary structure of the putative transport proteins was obtained from the deduced primary structure using the Gen-THREADER (http://bioinf.cs.ucl.ac.uk/psipred/).

In addition, an expression study of Eg-*pgp* genes (Eg-*pgp*1-a/b, Eg-*pgp*2, Eg-*pgp*3, Eg-*pgp*4 and Eg-*pgp*5) was carried out using the specific primers reported by Nicolao et al [22]. Total RNA extractions and RT-PCR from *E*. *granulosus* protoscoleces and metacestodes were performed as previously described Cumino et al [23]. To analyze the gene expression in pharmacologically treated and control parasites, cDNA was generated using 10 or 5 μg of total RNA from protoscoleces and metacestodes, respectively (with Superscript II reverse transcriptase -Invitrogen, Argentina—and Pfu DNA polymerase—Promega, USA). RT-PCR assays were carried out under the following conditions: 30 cycle PCRs of 94°C (30 s), 42°C (1 min), and 72°C (1 min) plus a single step at 72°C for 10 min, their products were analyzed and confirmed as it was previously described [23]. *E*. *granulosus* actin I (*act*I, GenBank accession number L07773) was used as a loading control [16, 24].

### Statistics

Data within experiments were compared and significance was determined using the student’s t test and the non-parametric Mann-Whitney test. All data were shown as arithmetic mean ± S.D. and *p* values are indicated in each assay.

### Accession numbers

The list of accession numbers mentioned in the text is shown below: GenBank, L07773: *Echinococcus granulosus* actin I (Eg-actI); GenBank, EUB61931: *E*. *granulosus* SLC22 B-6 (Eg-OCT-A); GenBank, EUB63000: *E*. *granulosus* SLC22 5 (Eg-OCT-B); GenBank, EUB61465: *E*. *granulosus* SLC22 5(Eg-OCT-C); GenBank, EUB64421: *E*. *granulosus* SLC22 (Eg-OCT-D); GenBank, EUB65032: *E*. *granulosus* SLC22 (Eg-SLC22-like); GenBank, O15245: *Homo sapiens* S22A1 (Hs-OCT1); GenBank, O15244: *H*. *sapiens* S22A2 (Hs-OCT2); GenBank, Q9H015: *H*. *sapiens* S22A4 (Hs-OCTN1); GenBank, O08966: *Mus musculus* S22A1 (Mm-OCT1); GenBank, O70577: *M*. *musculus* S22A2 (Mm-OCT2), GenBank, NP_062661: *M*. *musculus* S22A4 (Mm-OCTN1).

## Results

### Metformin exhibits *in vitro* anti-echinococcal effect and improves the efficacy of low concentrations of albendazole sulphoxide

**Fig 1:**
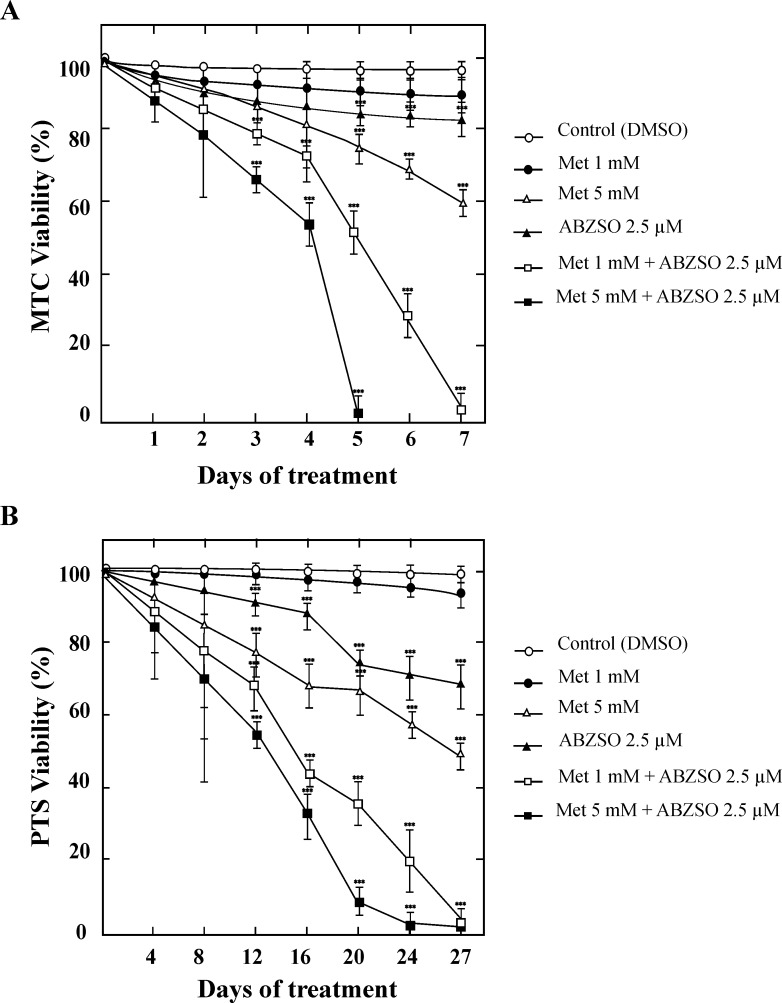
*In vitro* effect of metformin and its combination with low concentration of albendazole sulphoxide on viability of protoscoleces and metacestodes of *E*. *granulosus*. Viability of metacestodes (MTC) (A) and protoscoleces (PTS) (B) incubated for 7 and 27 days, respectively, with 1 and 5 mM of metformin alone (Met), 2.5 μM albendazole sulphoxide alone (ABZSO) and 1 and 5 mM Met plus 2.5 μM ABZSO in combination. Parasites incubated in culture medium containing DMSO served as controls. Data are the mean ± S.D. of three independent experiments. ***Statistically significant difference (*p* < 0.05) compared with control.

We have previously reported that Met exerts a dose-dependent effect on the viability of protoscoleces and metacestodes after 10 and 4 days of incubation, respectively. In addition, its combination with 15μM ABZSO resulted in a greater anti-echinococcal effect than the one observed for Met alone [16]. Here, we extended the study by using the minimum effective concentration of ABZSO (2.5μM) and evaluating the viability of protoscoleces and cysts over time. Administration of Met to cultured protoscoleces and metacestodes showed significant dose- and time-dependent killing effects. The mortality rate of both metacestodes and protoscoleces reached 100% during the combined chemotherapy with 1 mM Met and 2.5 μM ABZSO at days 7 and 27, respectively, whereas parasites treated with Met alone remained 90% (metacestodes) and 95% (protoscoleces) viable (Fig 1). Protoscolex mortality registered after incubations with 5 mM Met or 2.5 μM ABZSO for 27 days was only 50% and 30%, respectively (Fig 1B). As for metacestode viability, it decreased only 40% with 5 mM Met and 20% with 2.5 μM ABZSO in culture after 7 days (Fig 1A). These results suggest a statistically significant synergistic effect between Met and ABZSO from day 12 and day 3 against protoscoleces and metacestodes, respectively. Control parasites remained at least 99 ± 1.0% viable during the complete experiments.

### Metformin shows therapeutic effectiveness against fully-developed cyst

**Fig 2:**
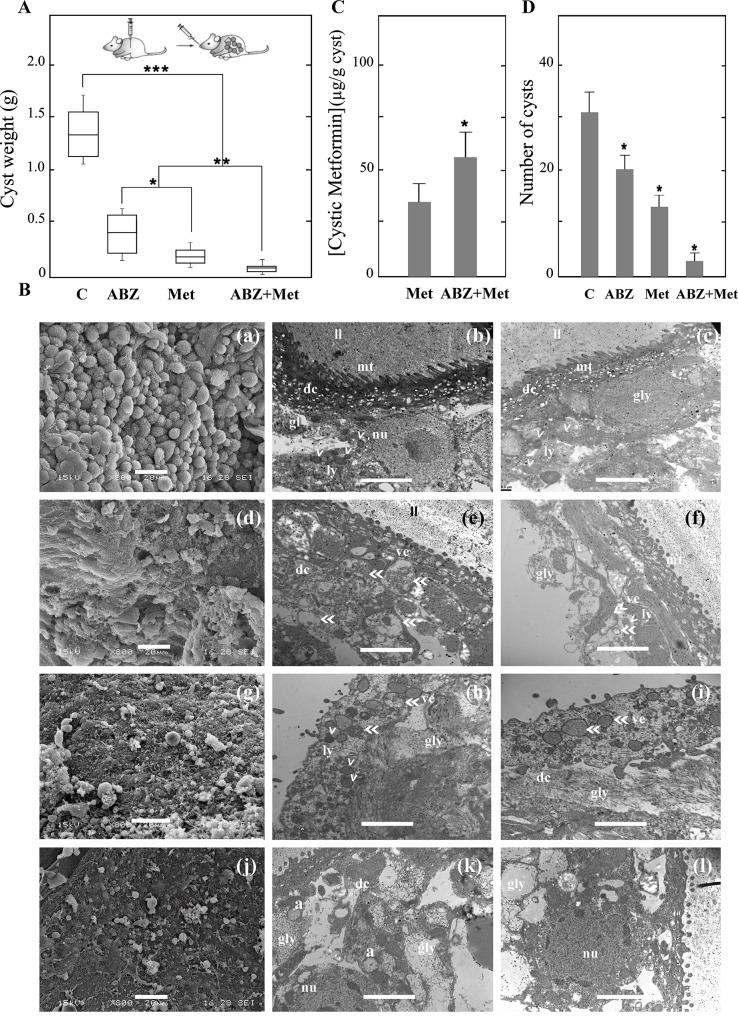
Therapeutic efficacy study in *E*. *granulosus* infected mice. (A) Box plots showing the comparative distribution of the weight (g) of cysts recovered from untreated mice (C) and treated with albendazole (ABZ, 5 mg/kg/d), metformin (Met, 50 mg/kg/d) and the combination of both drugs (ABZ+Met) for 60 days. The weight of cysts was significantly decreased upon all treatments compared with the control (***p < 0.01), but the decrease was more prominent in the group receiving the combined treatment than in those with either drug alone (**p < 0.05); in turn, weight reduction was greater with Met than with ABZ (*p < 0.2). (B) Representative SEM (a, d, g, j) and TEM (b, c, e, f, h, i, k, l) images of hydatid cysts recovered from untreated mice (a, b, c) or treated with Met (d, e, f), ABZ (g, h, i) and ABZ+Met (j, k, l). ll, laminated layer; mt, microtriches; dc, distal cytoplasm; gl, germinal layer; nu, nucleus; ly, lysosomes (arrowheads); ve: vesicles (double-headed arrow); gly, glycogen storage; a, autophagosomes. Bars indicate: 20 μm in (a, d, g, j), 1 μm in (b, e, f, h, I, k, l), and 0.2 μm in (c). (C) Intracystic concentrations of Met from cysts recovered from Met- or ABZ+Met-treated mice in the experiment indicated in (A). (D) Number of cysts obtained from untreated and pharmacologically treated mice as indicated in (A). *Statistically significant difference (*p* < 0.05) in (C) and (D).

To investigate the *in vivo* therapeutic effect of Met and ABZ, protoscoleces were intraperitoneally injected in CF1 mice and treated 4 months later by oral administration of vehicle, Met (50 mg/kg/day), ABZ (5 mg/kg/day) or the combination of Met plus ABZ (50 mg/kg/day plus 5 mg/kg/day) over a period of 60 days. All infected animals in this study developed hydatid cysts in their abdominal cavity. At 6 months p.i., every treatment from the therapeutic efficacy study (ABZ, Met and ABZ plus Met) resulted in a significant reduction (n = 10 *p* < 0.01) of the cyst weights compared to those obtained from untreated mice (1.450 ± 0.310 g) (Fig 2A). Cysts developed in mice belonging to the combined therapy group (0.08 ± 0.01 g for Met plus ABZ treatment) weighed significantly less (*p* < 0.05) than those from groups treated with each drug alone (0.200 ± 0.023 g for Met and 0.470 ± 0.040 g for ABZ treatments). Moreover, Met seemed to be more effective than ABZ when acting alone, since Met-treated mice cysts were reduced in weight (*p* < 0.2) in comparison with those recovered from ABZ-treated mice (Fig 2A). Nonetheless, both the number and size of cysts decreased after every treatment in contrast with the control, but particularly after treatment with Met and Met plus ABZ (30 ± 5 cysts for control, 20 ± 4 cysts for ABZ-, 12 ± 2 cysts for Met- and 5 ± 2 cysts for ABZ plus Met-treatments) (Fig 2D). No adverse effects or weight change were observed in mice.

In order to analyze the ultrastructural changes of cysts recovered from the different treatments, SEM and TEM studies were performed. Cysts from control mice at six months p.i. appeared turgid, with a massive amount of intact cells in germinal layers, according to SEM (Fig 2Ba and S1 Fig). By TEM analysis it was possible to determine that neither the external acellular laminated layer, the syncitial tegument with microtriches protruding into the laminated layer nor the germinal layer with intact tegumental cells showed any signs of ultrastructural alterations (Fig 2Bb and 2Bc and S1 Fig). Although, metacestodes collected from Met-treated mice displayed no marked reduction in the amount of germinal cells by SEM analysis (Fig 2Bd and S1 Fig), the TEM images showed a distorted tegument and several vesicles and lysosomes in the germinal layer, with reduction in glycogen storage (Fig 2Be and 2Bf and S1 Fig). In contrast, germinal layers of metacestodes obtained from ABZ- and ABZ-Met-treated mice exhibited partial and overall loss of cells, when analyzed by SEM, respectively (Fig 2Bg and 2Bj and S1 Fig). Moreover, TEM analysis of cysts obtained from ABZ-treated animals presented vesicles budding from the tegument with infiltration on the laminated layer and showed lack of microtriches (Fig 2Bh and 2Bi and S1 Fig). Meanwhile, metacestodes from ABZ-Met-treated mice evidenced complete disruption of the tissue containing autophagosomes (Fig 2Bk and 2Bl and S1 Fig).

Furthermore, Met concentration was measured in cysts obtained from Met- or Met plus ABZ-treated mice using hydatid liquid from cysts of untreated mice as negative control. (Fig 2C). Drug concentration was 35±7 μg/ g cyst in samples from animals treated with Met alone and 60±5 μg/ g cyst in those receiving both drugs.

### Metformin efficiently prevents hydatid cyst growth

**Fig 3:**
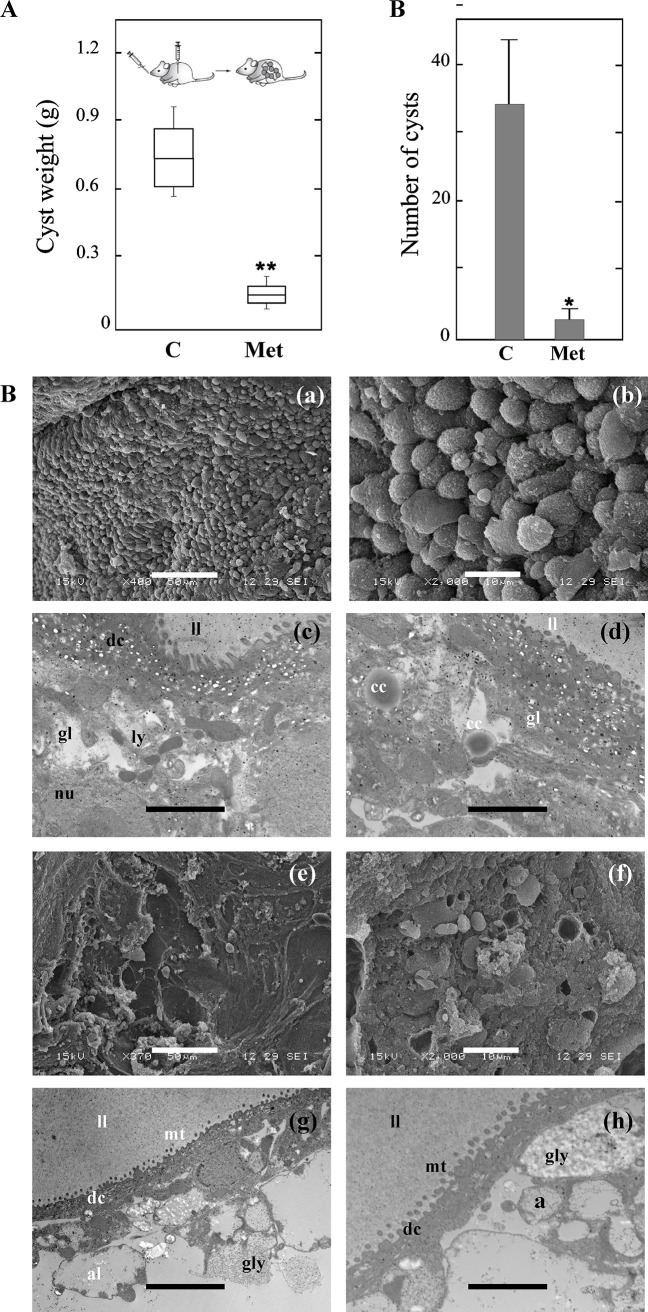
Chemoprophylactic activity of metformin during *E*. *granulosus* cyst development. (A) Box plot showing the comparative distribution of the weight (g) of cysts recovered from untreated (C) and Met-treated (Met, 50 mg/kg/d) mice. A significant cyst weight reduction (**p < 0.01) was achieved in treated animals. (B) Representative SEM (a, b, e, f) and TEM (c, d, g, h) images of hydatid cysts recovered from untreated control mice (a-d) compared with Met-treated mice (e-h). ll, laminated layer; mt, microtriches; dc, distal cytoplasm; gl, germinal layer; nu, nucleus; ly, lysosomes; gly, glycogen storage; a, autophagosomes; al, autophagolysosome; cc, calcareous corpuscles. Bars indicate: 50 μm in (a, e), 10 μm in (b, f) and 1 μm in (c, d, g, h). (C) Number of cysts obtained from untreated and Met-treated mice as indicated in (A).

To assess the potential chemopreventive effect of Met *in vivo*, the treatment (50 mg/kg/day) was initiated at the time of infection and followed during a period of 60 days. At 4 months p.i., mice were necropsied in order to remove the hydatid cysts from their abdominal cavities. All of the infected mice from the untreated group (10/10) developed metacestodes. However, the infection had not progressed in 2 out of the total of 10 mice treated with Met. Significant differences (*p* < 0.01) were registered in the weight of cysts obtained from untreated mice (0.730 ± 0.150 g) in comparison with those recovered from Met-medicated mice (0.131 ± 0.020 g, Fig 3A). Met-treated mice developed less and smaller cysts compared with untreated animals (35 ± 10 cysts for control and 4 ± 2 cysts for Met treatment) (Fig 3C). No adverse effects or weight change were observed in the group of treated mice.

Cysts removed from control mice were turgid and no changes in the ultrastructure of their germinal layers were detected by SEM (Fig 3Ba and 3Bb and S2 Fig). Moreover, their laminated and germinal layers showed typical features by TEM (Fig 3Bc and 3Bd and S2 Fig). Conversely, the ultrastructural analysis of metacestodes from Met-treated mice showed alterations in the germinal layer surface detected by SEM (Fig 3Be and 3Bf and S2 Fig) and revealed contraction of the distal cytoplasm as well as presence of autophagosomes and autophagolysosomes by TEM analysis (Fig 3Bg and 3Bh and S2 Fig). Interestingly, calcified cysts of 2–3 mm diameter revealed by TEM were found either in the liver or the surrounding abdominal area of 4 out of the total of 10 mice which were treated with Met.

Fibrosis was semiquantified using Image-J by histological examination of liver tissues stained with Masson’s trichrome, yet no significant differences were found between control and Met-treated samples (S3 Fig).

### Presence of putative organic cation transporters and Eg-*pgp* expression in *E*. *granulosus*

In order to evaluate the possible Met transporters that could account for intracystic drug accumulation, we investigated the occurrence of SLC22 family members in the *E*. *granulosus* larval stage. The SLC22 protein family includes several members which operate as transporters for organic cations (OCTs), organic zwitterions/cation (OCTNs) and organic anions (OATs). We previously identified two putative OCT/OCTNs (EgrG_001058900 and EgrG_000957000) in the *E*. *granulosus* genome [16], both of which displayed structural similarities and an identity range of 24–27% with the *H*. *sapiens* and *M*. *musculus* orthologs (S4 Fig). In this work, we completed the sequence analysis of all members of the SLC22 group found in the *Echinococcus* genome annotation. All putative SLC22 transporters show a membrane topology in accordance with the prototype carrier [27], which includes 10–12 α-helical transmembrane domains (TMDs), a large extracellular loop between TMDs 1 and 2 and an intracellular loop between TMDs 6 and 7 (S4 Fig). Due to the high identity of these predicted proteins with OCT/OCTN vertebrate orthologs, their genes were named as Eg-*oct*A, Eg-*oct*B, Eg-*oct*C and Eg-*oct*D (corresponding to EgrG_001058900, EgrG_000957000, EgrG_001099600 and EgrG_000994100, respectively). Only EgrG_000780900 showed an E-value higher than e^-25^ and thus its encoding gene was referred to as Eg-*slc22-like*. In the corresponding predicted proteins characteristic motifs of the amphiphilic solute facilitator (ASF) family (S[T/S]IVTE[W/F][D/N]LVC) were also identified together with the major facilitator superfamily (-MFS- 13-residue between TMDs 2 and 3: G-[RKPATY]-L-[GAS]-[DN]-[RK]-[FY]-G-R-[RKP]-[LIVGST]-[LIM]) and signature sequences after TMDs 10 and 11 as well (ELYPT and LP[D/E]TI, respectively) (S4 Fig).

Since Met and ABZSO have been suggested to be Pgp substrates [28, 29] and it has been shown that Met can modify Pgp expression in human cancer cell lines [30], we analyzed the transcriptional levels of the five Pgp isoforms described in both larval forms of *E*. *granulosus* [22]. No changes were observed when comparing transcript amounts between Met or ABZSO-treated and non-treated parasites (S5 Fig).

## Discussion

Human CE, though neglected, is a serious life-threatening zoonotic disease that occurs worldwide, which is recognized as a major public health problem [6]. Chemotherapeutic treatment success lies in the ability of the drug to affect the germinal layer and the intracystic protoscoleces at optimal concentrations for sufficient periods of time [31]. Currently, ABZ (which undergoes hepatic bioconversion into ABZSO) is the drug of preference to treat CE. However, since it is characterized by a low solubility in water, poor absorption in the intestinal tract and erratic penetration into hydatid cyst, its therapeutic effectiveness against CE is reduced [32]. Therefore, there is a dire need to find new drugs for the treatment of CE. In a previous report, the use of combined albendazole/nitazoxanide chemotherapy has been shown to exhibit anti-parasitic activity in an experimental model of murine alveolar echinococcosis [33]. However, neither nitazoxanide monotherapy nor albendazole-nitazoxanide combination therapies were effective against human alveolar echinococcosis [34]. In addition, drugs such as flubendazole and tamoxifen have been tested in the murine CE model as well as, showing a satisfactory success [35–37]. On the other hand, current studies have reported that Met, a hydrophilic drug which does not undergo hepatic metabolism, has an anti-proliferative effect on different cancer cell lines and several cancers in animal models [38–40]. In this line of evidence, we have previously found that Met by itself or in combination with ABZSO was effective against protoscoleces and metacestodes in *in vitro* assays [16]. In the present study, we report for the very first time that Met either by itself or together with ABZ has potential therapeutic effects and confers protection against the *E*. *granulosus* infection in an *in vivo* experimental model of secondary hydatidosis.

On the basis of our *in vitro* results, the effects of Met on protoscolex and metacestode viability were both dose- and time-dependent ([16] and this work). Moreover, the combined treatment with ABZSO (0.84 μg/ml) had a synergistic effect on the larval stage (Fig 1). The administration of a single 5 mg/kg/day oral dose of ABZ in mice [41] or 10–15 mg/kg/day in humans [42, 43], gives rise to a mean plasma concentration of 0.4–1 μg/ml of ABZSO after 2–4 h. Therefore, the here applied concentration of ABZSO is similar to the level reached in plasma on *in vivo* experimental models and corresponds to the minimum effective concentration of the drug [44–47]. On the other hand, the plasma Met concentration (0.5–4 μg/ml, about 3–25 μmol/l) recorded in mice 30 min after oral administration of a single bolus of 50 mg/kg is consistent with the maximum concentration observed in humans after consumption of a dose of about 8–25 mg/kg [9, 48]. In our *in vitro* experiments, Met concentrations used were a magnitude of order higher than plasma levels (Fig 1), but they are in the range used to examine the *in vitro* effects of the drug on cell metabolism and proliferation [49–51].

Regarding our *in vivo* assays, treatments with Met and ABZ have shown to have a marked therapeutic effect against murine experimental CE. Under these treatments the amount and weight of the recovered cysts were reduced, and ultrastructural changes were also observed (Fig 2D, 2A and 2B and S1 Fig) with Met being slightly more effective than ABZ. Nonetheless, the loss of cells from the germinal layer was more pronounced when using ABZ alone or combined compared to the use of Met alone (Fig 2Bd-i and S1 Fig). This can be attributed to the differences between both drugs in terms of biochemical actions. Given the fact that BZMs act on parasite tubulin and inhibit the assembly of microtubules, the cytological and cytostatic effects of ABZ could be associated to its interference with the correct functioning of the cellular cytoskeleton [52, 53]. As a consequence, this directly induces alterations in the vesicular transport in the absorptive/secretory tissues of helminths [52, 54]. In relation to this aspect, BZMs have shown to inhibit glucose uptake and to deplete glycogen stores, leading to starvation and death of the parasite [55, 56]. In addition, the loss of microtriches produced by ABZ (Fig 2Bh and 2Bi and S1 Fig) contributes to the reduction of nutrient uptake and subsequent anthelmintic effects [54]. On the other hand, Met inhibits complex I of the mitochondrial electron-transport chain, uncoupling ATP production and inducing energy stress in protoscoleces and germinal cells of the metacestode [16]. This mechanism of action would be enough to inhibit the growth of hydatid cysts, but not to induce cytotoxicity in germinal cells of metacestodes as ABZ does (Fig 2Bd-i and S1 Fig). The different cellular targets ABZ and Met would lead to synergistic effects, justifying the greater effectiveness of the combined treatment.

Moreover, the results of therapeutic efficacy assays in presence of Met showed drug accumulation in the cysts (300 μmol / kg of tissue) (Fig 2C) at similar levels to those found in liver tissue (200 μmol / kg of tissue) [9]. Although passive diffusion of Met through cell membranes is rather limited, its accumulation in the liver could generate a concentration gradient towards the hydatid cysts and, due to the high permeability of the cystic membranes to water [57, 58], the drug may penetrate by passive diffusion, as well as by facilitated transport through the OCTs [59]. Based on this, detection of higher levels of Met in cysts recovered from mice treated with the drug combination (Fig 2C) could also be attributed to the increase in permeability induced by the structural alterations of the metacestode laminar and germinal layers produced by ABZ. On the other hand, the intracystic accumulation of Met, is likely to be due to the presence of transporters involved in the uptake and efflux of Met in either the host [60] or the parasite (Fig 2C and S4 Fig). Met is a hydrophilic base which exists as an organic cation at physiological pH (p*K*a = 12.4) and requires SLC22 family proteins to be transported into the gut, the liver (OCT1) and the kidney (OCT1 and OCT2) of mammals [61]. In this work, we carried out an *in silico* analysis of sequences identified as members of the SLC22 family of *E*. *granulosus*. All five sequences presented the characteristic topology of these transporters, according to informatic predictions (S4 Fig) [62]. The occurrence and RNA levels of Eg-*oct*A (EUB61931) and Eg-*slc22*-like (EUB65032) have been reported in different *E*. *granulosus* stages by Zheng et al [63]. Further studies should be performed to evaluate the possible transport of Met in metacestodes via Eg-OCTs. A second transporter family implicated in the drug response involves the ABC (ATP-binding cassette) transporters, which include Pgp (an efflux pump that extrudes xenobiotics from cells), previously identified in *E*. *granulosus* [22]. Despite prior reports showing that Met is transported by Pgp [29] and that it inhibits Pgp transcriptional expression in human cancer cells [29], our findings indicated no differences in the transcriptional expression pattern of all Eg-Pgp isoforms in Met-treated protoscoleces and metacestodes (S5 Fig). Likewise, no transcriptional changes of these transporters were detected with ABZSO, a previously reported Eg-PgP substrate [22]. For this reason, Met accumulation in cysts (Fig 2C) could not be explained by changes in Pgp expression after treatment with Met or ABZSO.

In order to know the potential of Met to prevent a secondary hydatidosis caused by the release of protoscoleces during the surgery or the spontaneous cystic rupture, we carried out chemoprophylaxis assays. Met showed a remarkable inhibitory effect on cyst development (Fig 3A and 3C). In the first place, 20% of Met-treated mice did not develop any cyst, while the infection progressed in all untreated mice. The cystic development in treated mice is considered to occur from the originally injected protoscoleces that survived the therapy. In addition, Met not only reduced the number and weight of the cysts but also affected the integrity of germinal layer (Fig 3Be and 3Bf and S2 Fig). These results coincide with previously reported experimental chemopreventive studies using flubendazole and tamoxifen [37, 64]. However, it is important to consider the fact that Met has high anti-echinococcal efficacy and low toxicity, compared to those drugs. In this line of evidence, Met has been proposed as a safe and promising candidate for the prevention of colorectal cancer in normoglycemic patients [11, 13]. The mechanistic rationale for an anti-proliferative effect of Met is convincing, by antagonizing signaling pathways involved in cell division and migration, involving activation of the LKB1/AMPK pathway and subsequent inhibition of TORC1 pathway [65, 66]. We have previously identified TORC1 in the parasite, suggesting that the effects of Met against *E*. *granulosus* could be, at least partly, mediated by the AMPK-TORC1 pathway [16]. Additionally, activating AMPK, Met may oppose to the Warburg effect [38], a strategy acquired by *Echinococcus* germinal cells to cope with the high demand of both energy and intermediate metabolites under limited oxygen supply [67]. This metabolic switch from oxidative metabolism to glycolysis could confer to the germinal cells susceptibility to Met, as it has been described for tumor cells [68]. Besides, it has been reported that low doses of Met selectively kill cancer stem cells in different types of breast cancer [69]. This is consistent with the promising results of our chemoprophylaxis assay, since the drug could specifically affect germinal cells of the cyst. Conversely, BZMs have limited efficacy against undifferentiated germinal cells of this parasite [54, 70, 71]. Therefore, the combination of Met and ABZ may kill both stem cells and differentiated cells in the experimental echinococcosis model but this would have to be proven in further assays. On the other hand, it has been shown that Met has anti-angiogenic and anti-inflammatory activities, through which it could contribute to the interference in the establishment of the nematode parasite *Trichinella spiralis* [72]. Further studies must be carried out to evaluate the effects of Met in *E*. *granulosus* given that our preliminary study on the degree of fibrosis in the liver of infected mice was not enough to conclude on this point.

For clinical practice during long term treatment, Met meets the necessary criteria of a good candidate as chemopreventive and therapeutic agent such as safety, good compliance, cost-effectiveness and a clear mechanism of action [13]. Its most frequent adverse events are mild and transient gastrointestinal symptoms [73] as well as rare incidence of lactic acidosis [74, 75]. In conclusion, in this report we provide evidence into the potential benefits of Met as a new treatment option for CE, and these observations provide the impetus to evaluate the role of Met in the development of other helminths. These findings enhance the importance of carrying out further studies to determine the significance of the use of Met in relation to hydatidosis in humans.

## Supporting information

S1 Fig Ultrastructural characteristics of fully-developed cysts removed from mice under therapeutic efficacy study. Representative SEM (a, b, e, f, i, j, m, n) and TEM (c, d, g, h, k, l, o, p) images of hydatid cysts recovered from untreated mice (a-d) or treated with Met (e-h), ABZ (i-l) and ABZ+Met (m-p). ll, laminated layer; mt, microtriches; dc, distal cytoplasm; gl, germinal layer; ve: vesicles (double-headed arrow); gly, glycogen storage; cc: calcareous corpuscles. Bars indicate: 50 μm in (e, m), 20 μm in (a, i, n), 10 μm in (b, f, j), and 1 μm in (c, d, g, h, k, l, o, p).
(TIF)

S2 Fig Ultrastructural characteristics of cysts in development removed from mice under preventive efficacy study. Representative SEM (a, b, e, f) and TEM (c, d, g, h) images of hydatid cysts recovered from untreated control mice (a-d) compared with Met-treated mice (e-h). ll, laminated layer; mt, microtriches; mit: mitochondria; dc, distal cytoplasm; gl, germinal layer; nu, nucleus; ly, lysosomes; gly, glycogen storage; al, autophagolysosome; Bars indicate: 20 μm in (a, e), 10 μm in (b, f) and 1 μm in (c, d, g, h).
(TIF)

S3 Fig Evaluation of hepatic inflammation and fibrosis caused by *E*. *granulosus* in pharmacologically treated and untreated mice. **(A)** Histological examination of liver tissues from 6 months *E*. *granulosus-*infected mice after oral administration of vehicle (C), metformin (Met, 50 mg/kg/day), albendazole (ABZ, 5 mg/kg/day) and their combination (Met+ABZ, 50 mg/kg/day + 5 mg/kg/day) during 60 days. The inflammatory response (double black arrowhead) was assessed by hematoxylin and eosin (H&E) staining (a-d), while fibrosis (double black arrowhead) was assessed using Masson´s trichrome (Masson TC) staining (e-h). **(B)** Histological examination of liver tissues from 4 months *E*. granulosus-infected mice after oral administration of vehicle (C) and metformin (Met, 50 mg/kg/day) during 60 days. The inflammatory response (double black arrowhead) was assessed by hematoxylin and eosin (H&E) staining (a, b) while fibrosis (double black arrowhead) was assessed using Masson´s trichrome (Masson TC) staining (c, d). Although the treatment with Met apparently reduced the inflammatory response and the hepatic fibrosis around of cyst, statistically significant differences were not detected in comparison with the control (A, B). Since the distribution, the development stages as well as the number of hepatic cysts were different among treatments (C and ABZ treatment showed higher number of hepatic cysts compared to Met and Met+ABZ treatments), the degree of inflammatory response and hepatic fibrosis could not be attributed only to the treatments with the drugs.
(TIF)

S4 Fig Identification of the genes encoding solute carrier family 22 (SLC22) proteins in *E*. *granulosus* and multiple sequence alignment of their deduced amino acid sequences. (A) Comparison of *E*-values between the protein sequences reported for *E*. *granulosus* SLC22s in GeneDB (systematic names: EgrG_001058900, EgrG_000957000, EgrG_001099600, EgrG_000994100, EgrG_000780900) or in GenBank (accession numbers: EUB61931, EUB63000, EUB61465, EUB64421 and EUB65032) and the sequences corresponding to SLC22A isoform 1 (organic cation transporter 1 -OCT1-) for *Homo sapiens* (GenBank accession number O15245) and *Mus musculus* (GenBank accession number O08966), SLC22A isoform 2 (OCT2) for *H*. *sapiens* (GenBank accession number O15244) and *M*. *musculus* (GenBank accession number O70577) and SLC22A isoform 4 (Organic cation/carnitine transporter 1 -OCTN1-) for *H*. *sapiens* (GenBank accession number Q9H015) and *M*. *musculus* (GenBank accession number NP_062661). The “Protein size” column indicates the total number of amino acid residues in the sequences, and the “TM Domain” column gives the number of transmembrane domains of each sequence predicted by SACS MEMSAT2 Transmembrane Prediction Program. (B) Amino acid sequence comparison between *Echinococcus* SLC22s and mammalian orthologs. Consensus is indicated in the last line, total (uppercase letter), partial (lowercase letter), conservative changes (numeral), absence of consensus (dots) and gaps introduced to maximize the alignment (dashes). Sequences present 12 transmembrane domains (TMD1-12) (gray boxes), conserved cysteins (black arrowheads) that could be involved in the oligomerization and glycosylation sites (black circles) between TMDs 1 and 2, conserved phosphorylation sites for protein kinases between TMDs 6 and 7 (asterisks), an ASF motif (orange box), a MSF motif (green box), and key residues identified by mutagenesis assays as very important to preserve the high transport of metformin in humans (R61, G401 and G465, light blue boxes) [17]. GenBank accession numbers for the OCT1/OCTN1 proteins are: Mm, *Mus musculus* (O08966 and NP_062661); Eg, *Echinococcus granulosus* (EUB61931, EUB63000, EUB61465, EUB64421 and EUB65032).
(TIF)

S5 Fig Transcriptional expression of *E*. *granulosus* Pgp isoforms in pharmacologically treated and untreated parasites. Reverse Transcription (RT)-PCR analysis from total RNA of protoscoleces (PTS) and metacestodes (MTC) incubated for 48 h under control conditions (C) and treated with 5 mM metformin (Met) or 2.5 μM albendazole sulphoxide (ABZSO). Amplification of Eg-*actin* I (*act*I) was used as a loading control. Molecular sizes of amplicons are indicated with arrowheads.
(TIF)
