# Correction: Metformin exhibits preventive and therapeutic efficacy against experimental cystic echinococcosis

**DOI:** 10.1371/journal.pntd.0005474

**Published:** 2017-03-22

**Authors:** Julia A. Loos, Valeria A. Dávila, Christian Rodriguez Rodrigues, Romina Petrigh, Jorge A. Zoppi, Fernando A. Crocenzi, Andrea C. Cumino

The third author’s name is spelled incorrectly. The correct name is: Christian Rodriguez Rodrigues. The correct citation is: Loos JA, Dávila VA, Rodriguez Rodrigues C, Petrigh R, Zoppi JA, Crocenzi FA, et al. (2017) Metformin exhibits preventive and therapeutic efficacy against experimental cystic echinococcosis. PLoS Negl Trop Dis 11(2): e0005370. doi:10.1371/journal.pntd.0005370
