# Supplementary figures and images for: Metformin exhibits preventive and therapeutic efficacy against experimental cystic echinococcosis
Source: PLoS Negl Trop Dis. 2017 Feb 9;11(2):e0005370. doi: 10.1371/journal.pntd.0005370 (PMC5321462; doi:10.1371/journal.pntd.0005370)

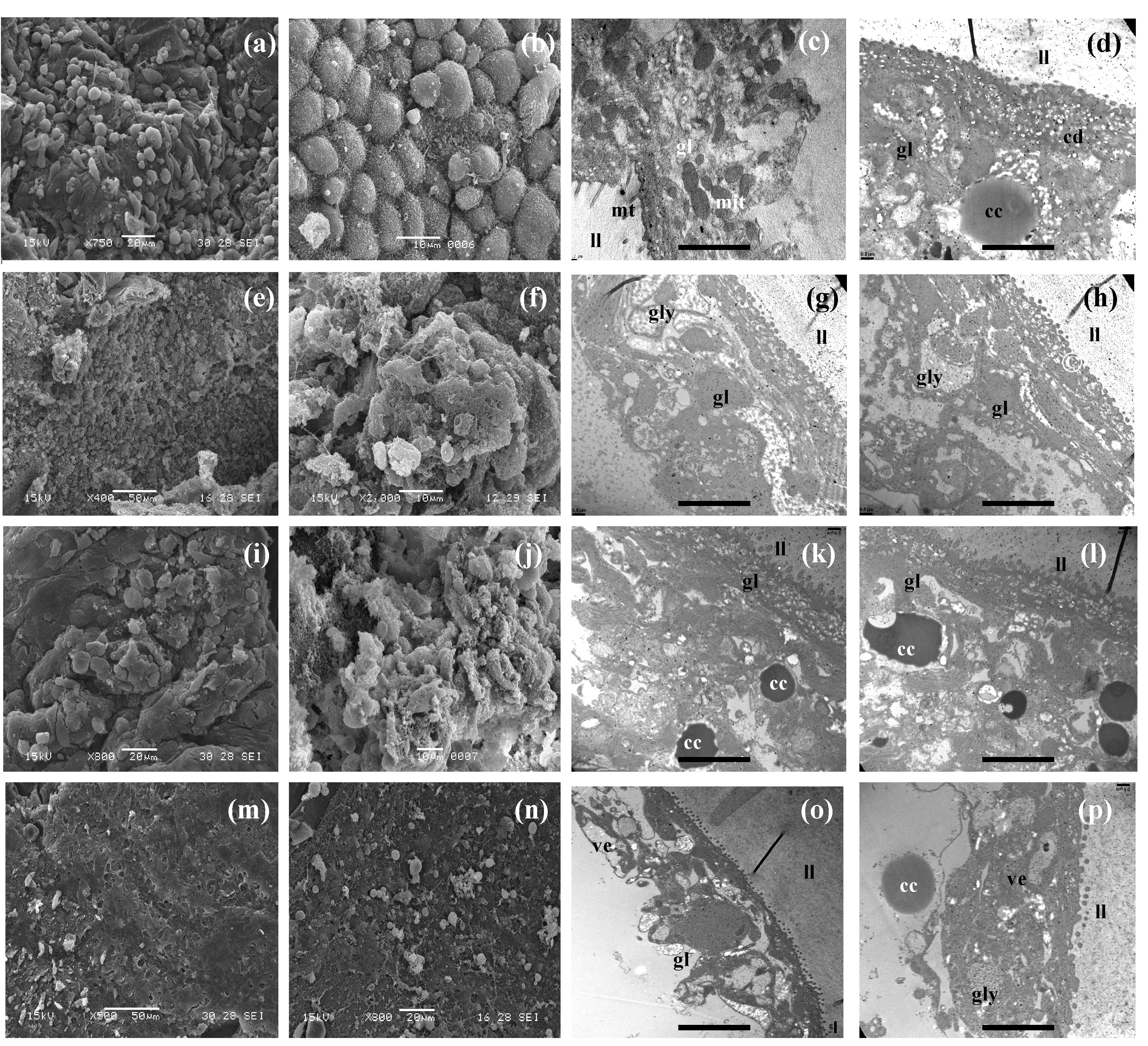

Supplement: S1 Fig — Representative SEM (a, b, e, f, i, j, m, n) and TEM (c, d, g, h, k, l, o, p) images of hydatid cysts recovered from untreated mice (a-d) or treated with Met (e-h), ABZ (i-l) and ABZ+Met (m-p). ll, laminated layer; mt, microtriches; dc, distal cytoplasm; gl, germinal layer; ve: vesicles (double-headed arrow); gly, glycogen storage; cc: calcareous corpuscles. Bars indicate: 50 μm in (e, m), 20 μm in (a, i, n), 10 μm in (b, f, j), and 1 μm in (c, d, g, h, k, l, o, p). (TIF) [file pntd.0005370.s001.tif]

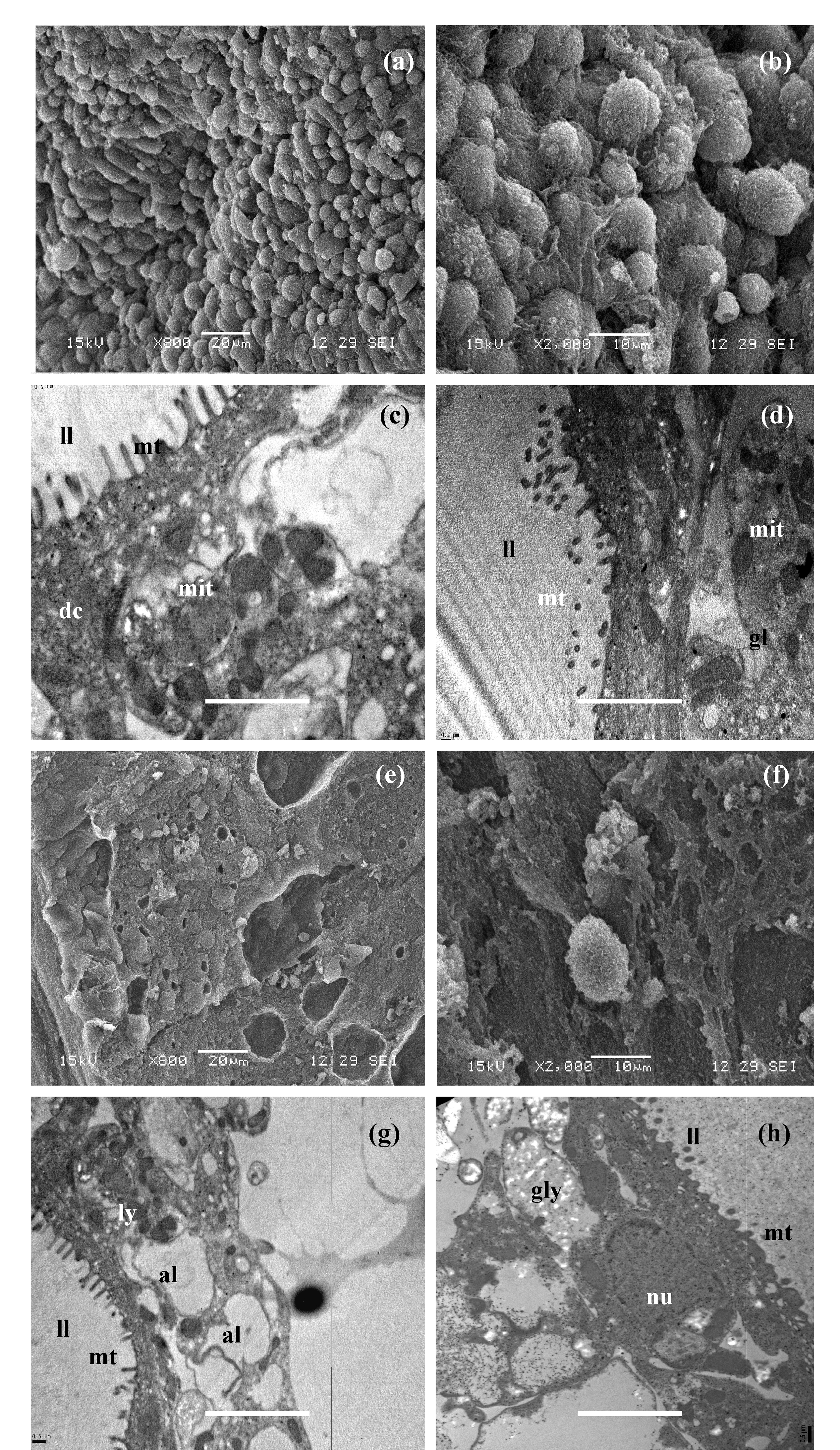

Supplement: S2 Fig — Representative SEM (a, b, e, f) and TEM (c, d, g, h) images of hydatid cysts recovered from untreated control mice (a-d) compared with Met-treated mice (e-h). ll, laminated layer; mt, microtriches; mit: mitochondria; dc, distal cytoplasm; gl, germinal layer; nu, nucleus; ly, lysosomes; gly, glycogen storage; al, autophagolysosome; Bars indicate: 20 μm in (a, e), 10 μm in (b, f) and 1 μm in (c, d, g, h). (TIF) [file pntd.0005370.s002.tif]

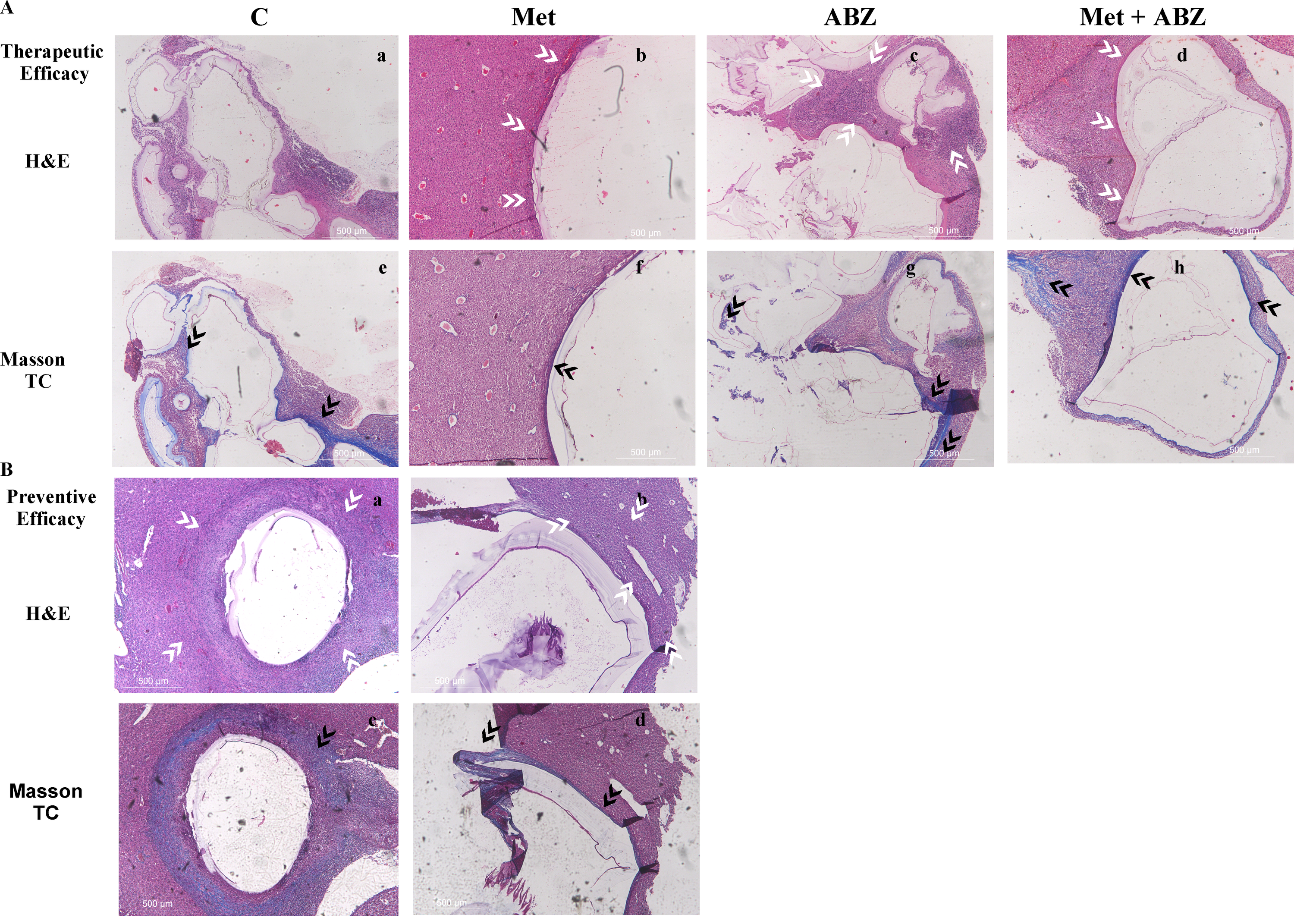

Supplement: S3 Fig — (A) Histological examination of liver tissues from 6 months E. granulosus-infected mice after oral administration of vehicle (C), metformin (Met, 50 mg/kg/day), albendazole (ABZ, 5 mg/kg/day) and their combination (Met+ABZ, 50 mg/kg/day + 5 mg/kg/day) during 60 days. The inflammatory response (double black arrowhead) was assessed by hematoxylin and eosin (H&E) staining (a-d), while fibrosis (double black arrowhead) was assessed using Masson´s trichrome (Masson TC) staining (e-h). (B) Histological examination of liver tissues from 4 months E. granulosus-infected mice after oral administration of vehicle (C) and metformin (Met, 50 mg/kg/day) during 60 days. The inflammatory response (double black arrowhead) was assessed by hematoxylin and eosin (H&E) staining (a, b) while fibrosis (double black arrowhead) was assessed using Masson´s trichrome (Masson TC) staining (c, d). Although the treatment with Met apparently reduced the inflammatory response and the hepatic fibrosis around of cyst, statistically significant differences were not detected in comparison with the control (A, B). Since the distribution, the development stages as well as the number of hepatic cysts were different among treatments (C and ABZ treatment showed higher number of hepatic cysts compared to Met and Met+ABZ treatments), the degree of inflammatory response and hepatic fibrosis could not be attributed only to the treatments with the drugs. (TIF) [file pntd.0005370.s003.tif]

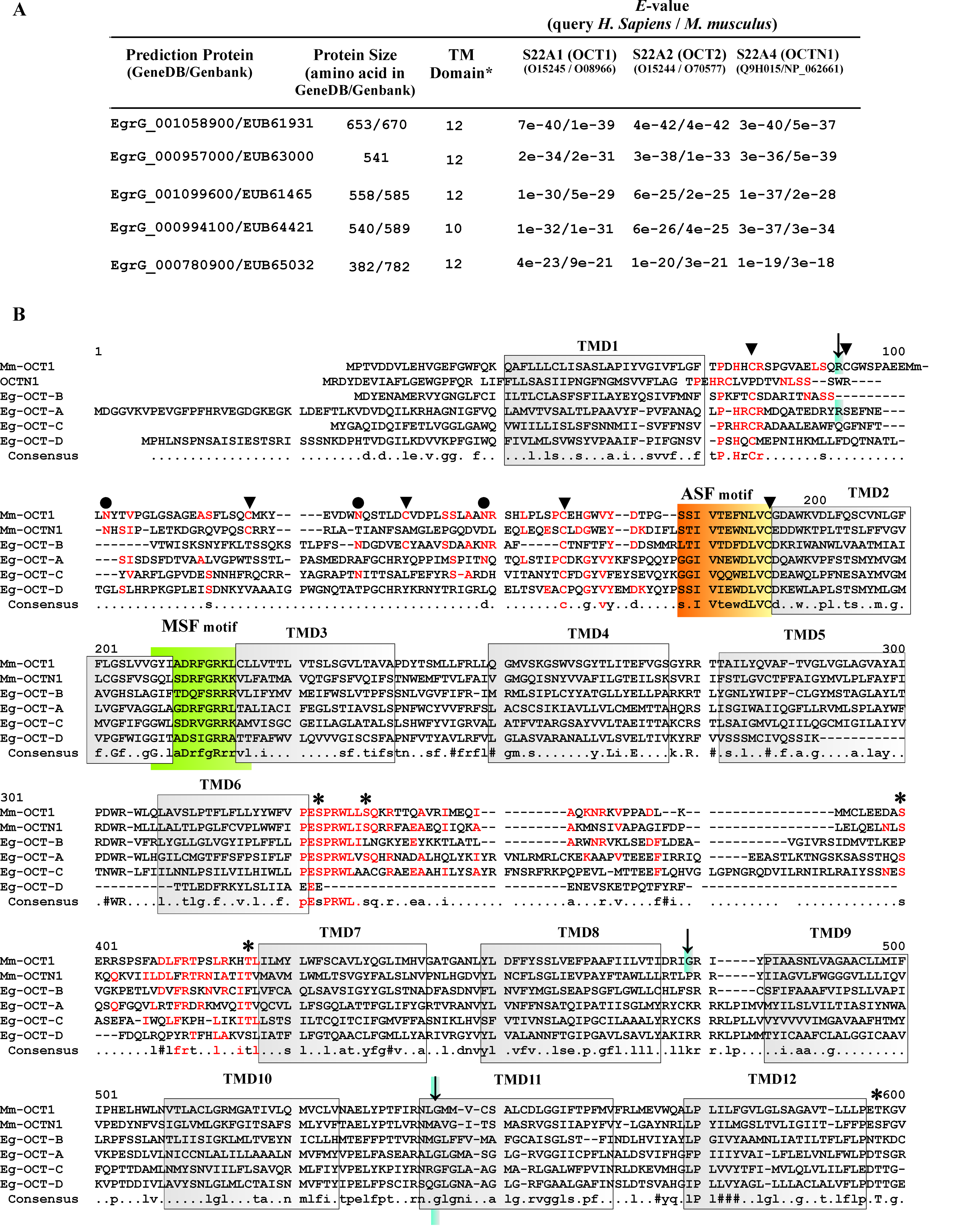

Supplement: S4 Fig — (A) Comparison of E-values between the protein sequences reported for E. granulosus SLC22s in GeneDB (systematic names: EgrG_001058900, EgrG_000957000, EgrG_001099600, EgrG_000994100, EgrG_000780900) or in GenBank (accession numbers: EUB61931, EUB63000, EUB61465, EUB64421 and EUB65032) and the sequences corresponding to SLC22A isoform 1 (organic cation transporter 1 -OCT1-) for Homo sapiens (GenBank accession number O15245) and Mus musculus (GenBank accession number O08966), SLC22A isoform 2 (OCT2) for H. sapiens (GenBank accession number O15244) and M. musculus (GenBank accession number O70577) and SLC22A isoform 4 (Organic cation/carnitine transporter 1 -OCTN1-) for H. sapiens (GenBank accession number Q9H015) and M. musculus (GenBank accession number NP_062661). The “Protein size” column indicates the total number of amino acid residues in the sequences, and the “TM Domain” column gives the number of transmembrane domains of each sequence predicted by SACS MEMSAT2 Transmembrane Prediction Program. (B) Amino acid sequence comparison between Echinococcus SLC22s and mammalian orthologs. Consensus is indicated in the last line, total (uppercase letter), partial (lowercase letter), conservative changes (numeral), absence of consensus (dots) and gaps introduced to maximize the alignment (dashes). Sequences present 12 transmembrane domains (TMD1-12) (gray boxes), conserved cysteins (black arrowheads) that could be involved in the oligomerization and glycosylation sites (black circles) between TMDs 1 and 2, conserved phosphorylation sites for protein kinases between TMDs 6 and 7 (asterisks), an ASF motif (orange box), a MSF motif (green box), and key residues identified by mutagenesis assays as very important to preserve the high transport of metformin in humans (R61, G401 and G465, light blue boxes) [17]. GenBank accession numbers for the OCT1/OCTN1 proteins are: Mm, Mus musculus (O08966 and NP_062661); Eg, Echinococcus granulosus (EUB61931, EUB63000, EUB61465, EUB64421 [file pntd.0005370.s004.tif]

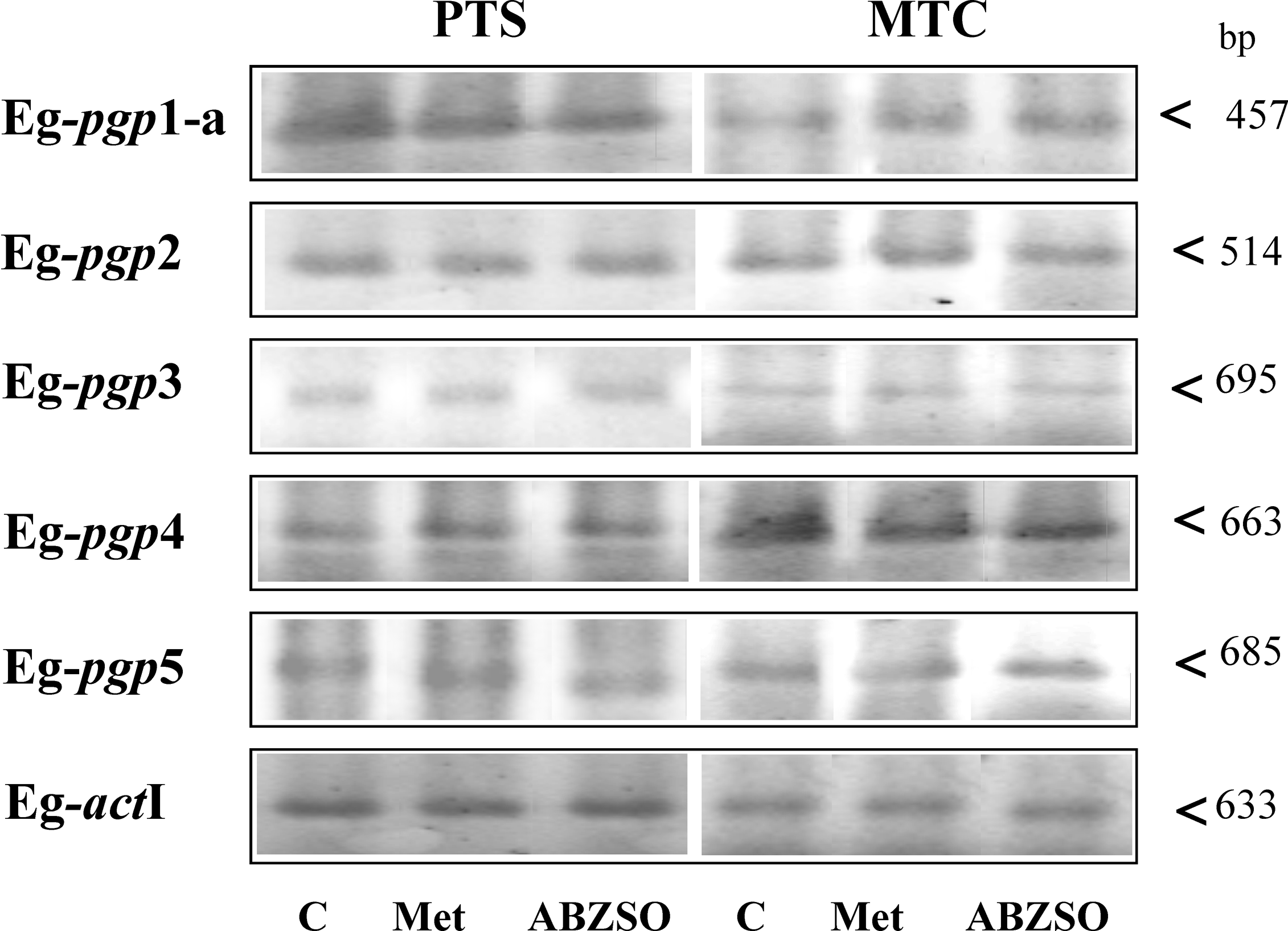

Supplement: S5 Fig — Reverse Transcription (RT)-PCR analysis from total RNA of protoscoleces (PTS) and metacestodes (MTC) incubated for 48 h under control conditions (C) and treated with 5 mM metformin (Met) or 2.5 μM albendazole sulphoxide (ABZSO). Amplification of Eg-actin I (actI) was used as a loading control. Molecular sizes of amplicons are indicated with arrowheads. (TIF) [file pntd.0005370.s005.tif]
